# New strategies for base frame fabrication in microtia reconstruction

**DOI:** 10.1038/s41598-021-95613-3

**Published:** 2021-08-05

**Authors:** Yiyuan Li, Datao Li, Zhicheng Xu, Ruhong Zhang, Qun Zhang, Feng Xu, Xia Chen

**Affiliations:** Department of Plastic and Reconstructive Surgery, Shanghai 9th People’s Hospital, Shanghai Jiao Tong University School of Medicine, 639 Zhi Zao Ju Road, Shanghai, 200011 People’s Republic of China

**Keywords:** Signs and symptoms, Therapeutics

## Abstract

The base frame provides a stable support for the helix, antihelix, and tragus–antitragus complex in microtia reconstruction, and this support is vital to attain a highly defined outline for a reconstructed auricle. The success of base frame sculpting depends on appropriate treatment of the cartilage, mainly the sixth and seventh costal cartilages, which may have different characteristics. The aim of this study was to demonstrate the relevant details for base frame fabrication under various scenarios. Between 2016 and 2019, a total of 352 patients with microtia underwent autologous auricular reconstruction. Concerning the different sizes and characteristics of the costal cartilage used for the base frame reconstruction, we describe the related methods for fabrication and introduce corresponding strategies for proper management. We found that 90% of the patients responded at follow-up, and 76% of them were satisfied with the cosmetically refined auricle with harmonious integrity. The elaborate design and appropriate utilization of costal cartilage for base frame sculpting is one of the most significant and fundamental processes in microtia reconstruction. It contributes to achieving a clearly defined outline of the auricle with harmonious integrity, which is as important as the other projected subunits.

## Introduction

Ear reconstruction, in some sense, is a highly complicated process of reproducing a harmonious concave–convex auricular contour, which relies mainly on the successful fabrication of such intricate protruding subunits as the helix, antihelix, and tragus–antitragus complex. Significant improvements in framework fabrication have been made in recent decades^1–6^. It is noteworthy that the base frame is a reliable foundation for the important structures mentioned above, or in some contexts, it may integrate into and become a part of them. Therefore, base frame sculpting is of great importance in microtia reconstruction, and it is no less important than the other projected subunits. However, the different characteristics that arise because of the complicated conditions of the sixth and seventh costal cartilage are among the most demanding challenges in base frame fabrication. The aim of this article is to demonstrate the relevant details for base frame fabrication in various scenarios and to introduce corresponding strategies for proper management.

## Methods

### Patients and methods

From October 2016 to December 2019, a total of 352 patients (age range, 6–52 years; 237 male patients and 115 female patients) underwent two-stage reconstruction for microtia (right-sided, 229; left-sided 102; bilateral, 21) with autogenous costal cartilage by the modified Brent and Nagata’s techniques (Table 1), as described previously^7–10^.

**Table 1:** Clinical data of the 352 microtia patients.

Characteristic	No. of patients (%)
**Gender**	
Male	237 (67.3)
Female	115 (32.7)
Age (years)	
6–10	83 (23.6)
11–15	122 (34.6)
16–20	80 (22.7)
21–52	67 (19.1)
**Affected side**	
Right	229 (65.1)
Left	102 (28.9)
Bilateral	21 (6.0)
**Microtia type**	
Lobule	259 (73.6)
Concha	93 (26.4)

This study was approved by the Ethics Committee of Shanghai Ninth People’s Hospital affiliated with the Shanghai Jiao Tong University School of Medicine (reference no. 2016-135-T84). All methods were carried out in accordance with the relevant guidelines and regulations. Informed consent was obtained from all participants and their legal guardians. Consent to publish from the participants and legal guardians of the minor participants for the mentioned case report was obtained.

### Harvesting the rib cartilage

We prefer to harvest three costal cartilages (the sixth, seventh, and eighth) from the contralateral chest; when necessary, the ninth costal cartilage is also prepared for use. The sixth and seventh costal cartilages are for the base frame and tragus–antitragus complex reconstruction. The antihelix could be from the residual part of the seventh or eighth costal cartilage or the ninth costal cartilage according to the variable conditions of each case. The eighth costal cartilage is used to form the helix and crus helicis.

### Base frame fabrication

In general, the sixth and seventh costal cartilages are mainly used for base frame fabrication. Our process has gradually evolved in response to our experiences, and different approaches to fabricating the base frame and the relative projected subunits have been established to attain relatively excellent results (Table 2).

**Table 2:** Summary of the base frame fabrication with individualized conditions of the ribs in microtia reconstruction.

Related structures	Individualized condition	Procedure	Type
Base frame	Integral 6th and 7th ribs with proper width and thickness	General procedure	A1
Base frame	Separated 6th and 7th ribs with proper width and thickness	Steel wires fixation	A2
Base frame; antihelical complex	Integral and thick 6th and 7th ribs with proper width	Direct carving on base frame	B
Base frame; helix	Narrow 6th rib with proper thickness	Cartilage block broadening	C1
Base frame; helix	Thick 7th rib and short 8th rib	Lower edge of 7th rib plus short 8th rib	C2
Base frame; helix	Calcified or brittle 8th rib	Edge of 6th and 7th ribs as helix substitution	C3
Base frame; tragus–antitragus complex	Narrow 7th rib with proper thickness	Cartilage block broadening	D
Base frame; ear lobule	No residual tissue utilized as lobule	Thickened lower part of 7th rib plus cartilage block at the bottom	E

### Part A general procedure of the base frame fabrication

**Figure 1:**
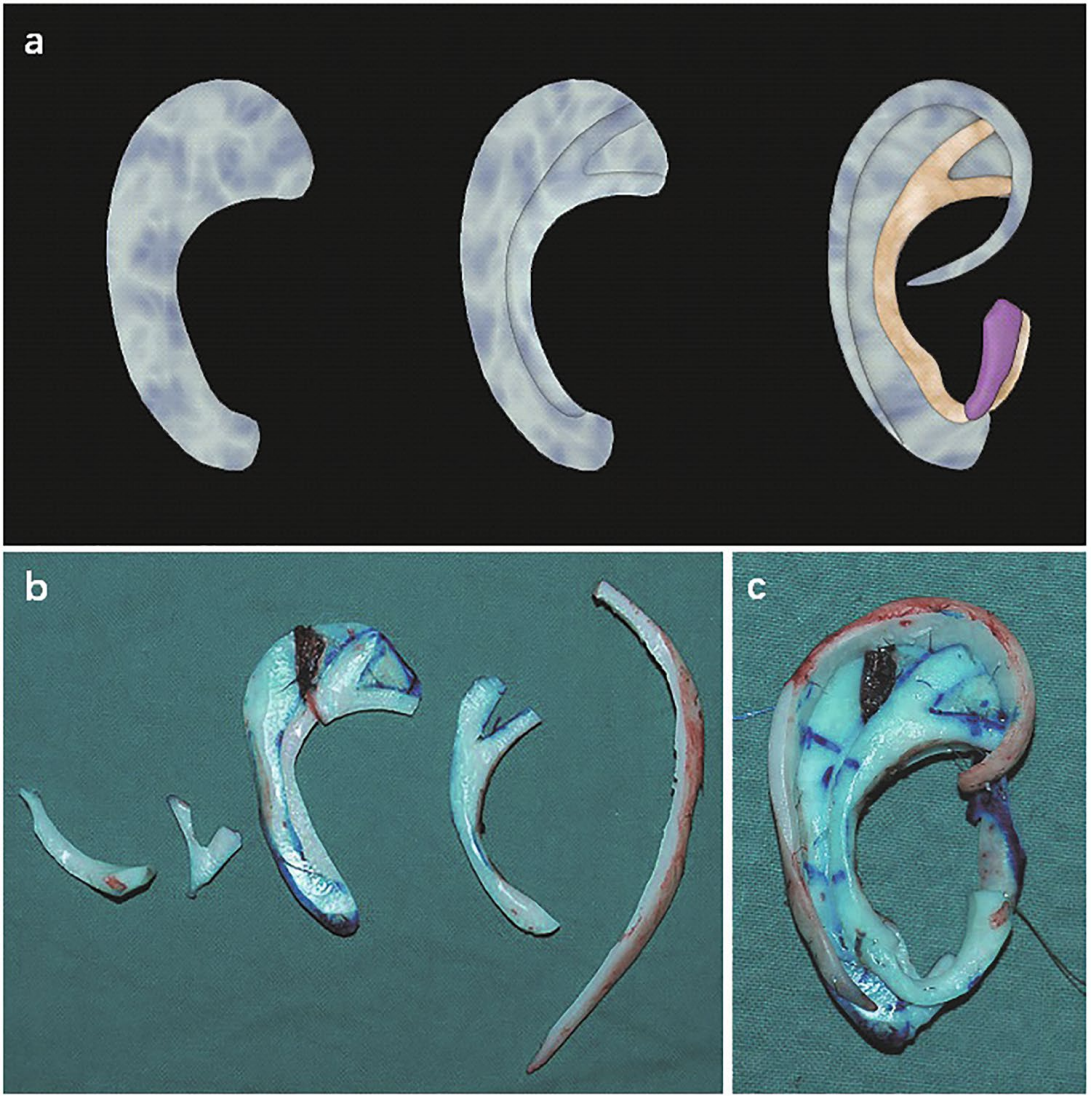
Schematic representation of the general procedure of the base frame fabrication (Type A1). (**a**) The base frame is well constructed with completely integral synchondrosis and proper thick cartilage. (**b**) A groove is carved into the base frame to accommodate the Y-shaped antihelix complex. (**c**) Completed framework ready for reconstruction.

We found that the base frame can be well constructed if the synchondrosis of the two ribs is completely integral and either of the costal cartilages is broad enough to match the template from the contralateral normal side. In our experience, we consider it appropriate to keep the average thickness of the base frame between 4 and 5 mm and to reduce it by 1–2 mm in diameter compared to the normal side, considering the thickness of the skin. Moreover, a groove is often carved into the base frame to accommodate the antihelix. Furthermore, the edge of the dorsal part of the base frame should be sculpted as smoothly as possible. As described above, this approach yields a stable base frame with the proper thickness and a smooth edge (Fig. 1, type A1).

**Figure 2:**
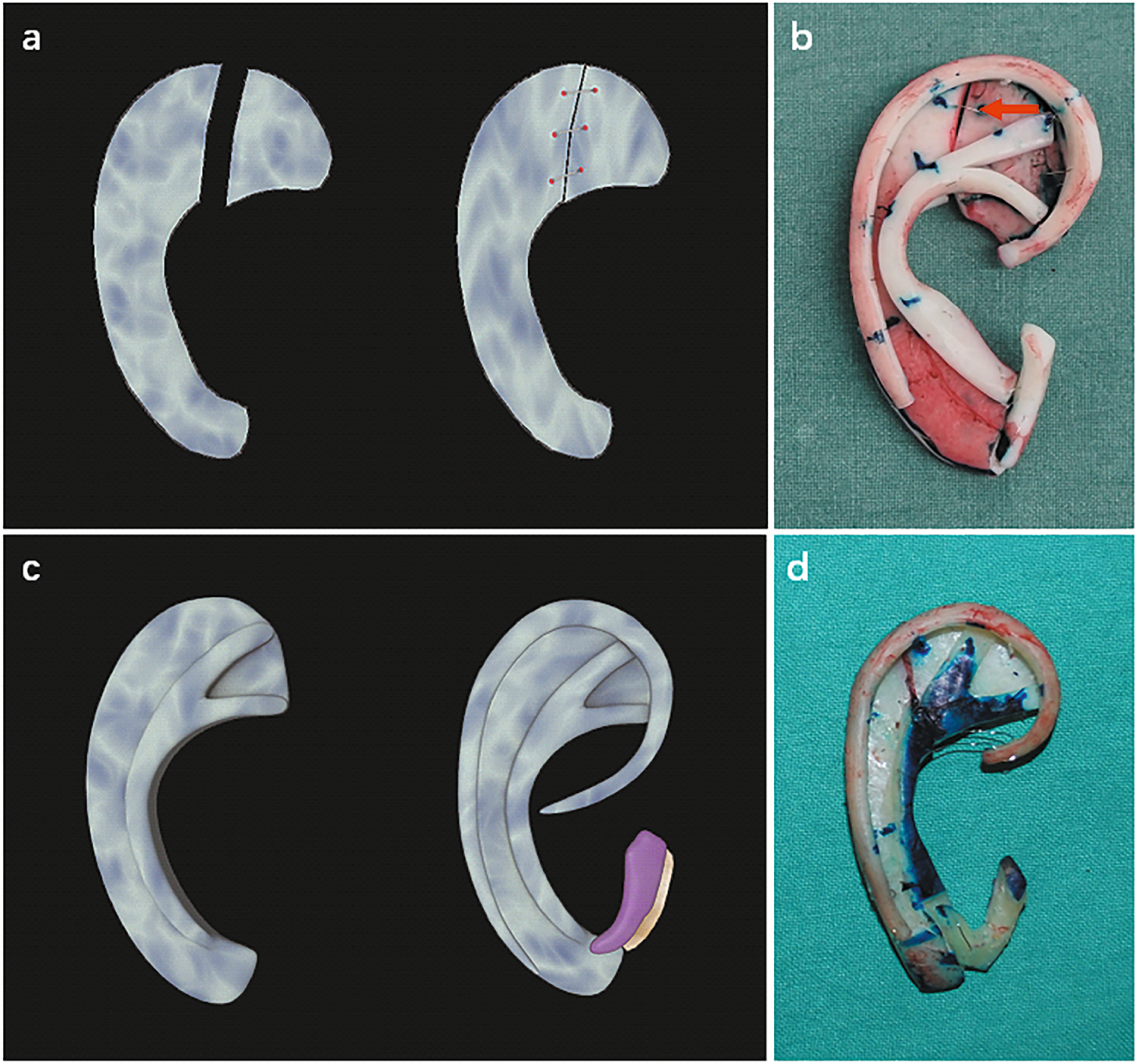
Schematic representation of the base frame fabrication with separate synchondrosis (**a**,**b**) and adequately thick cartilage (**c**,**d**). (**a**) The separate upper part of the base frame is fixed by stainless steel wires. (**b**) The firmly fixed three-dimensional framework. The red arrow shows the connection point. (**c**) The Y-shaped antihelical complex carved directly from the base frame. (**d**) The completed three-dimensional framework.

However, the synchondrosis is frequently separate or connected incompletely. The base frame is unstable and cannot support the structures of the antihelix complex and helix fixed on it afterward. Therefore, the separate upper part of the base frame is fixed by a stainless-steel wire at a distance of 2.5 mm from the edge. This is sufficient to ensure the stability and firmness of the base frame by two or three fixation points (Fig. 2a,b, red arrows in Fig. 5a–e, type A2).

### Part B base frame and antihelical complex fabrication

As mentioned in Part A, notching often occurs when the synchondrosis is not firmly connected. Thus, a Y-shaped antihelix is commonly added to hide the rough appearance and thus improve the stability of the framework and to give prominence to the smooth contour of the antihelix.

If the cartilage is thick enough, and it is commonly more than 5 mm in thickness, and simultaneously has no notch at the synchondrosis, the Y-shaped antihelical complex may be carved directly from the base frame. This procedure may be suitable for patients with strong cartilage (Figs. 2c,d, 5a,b, type B).

### Part C base frame and helix fabrication

**Figure 3:**
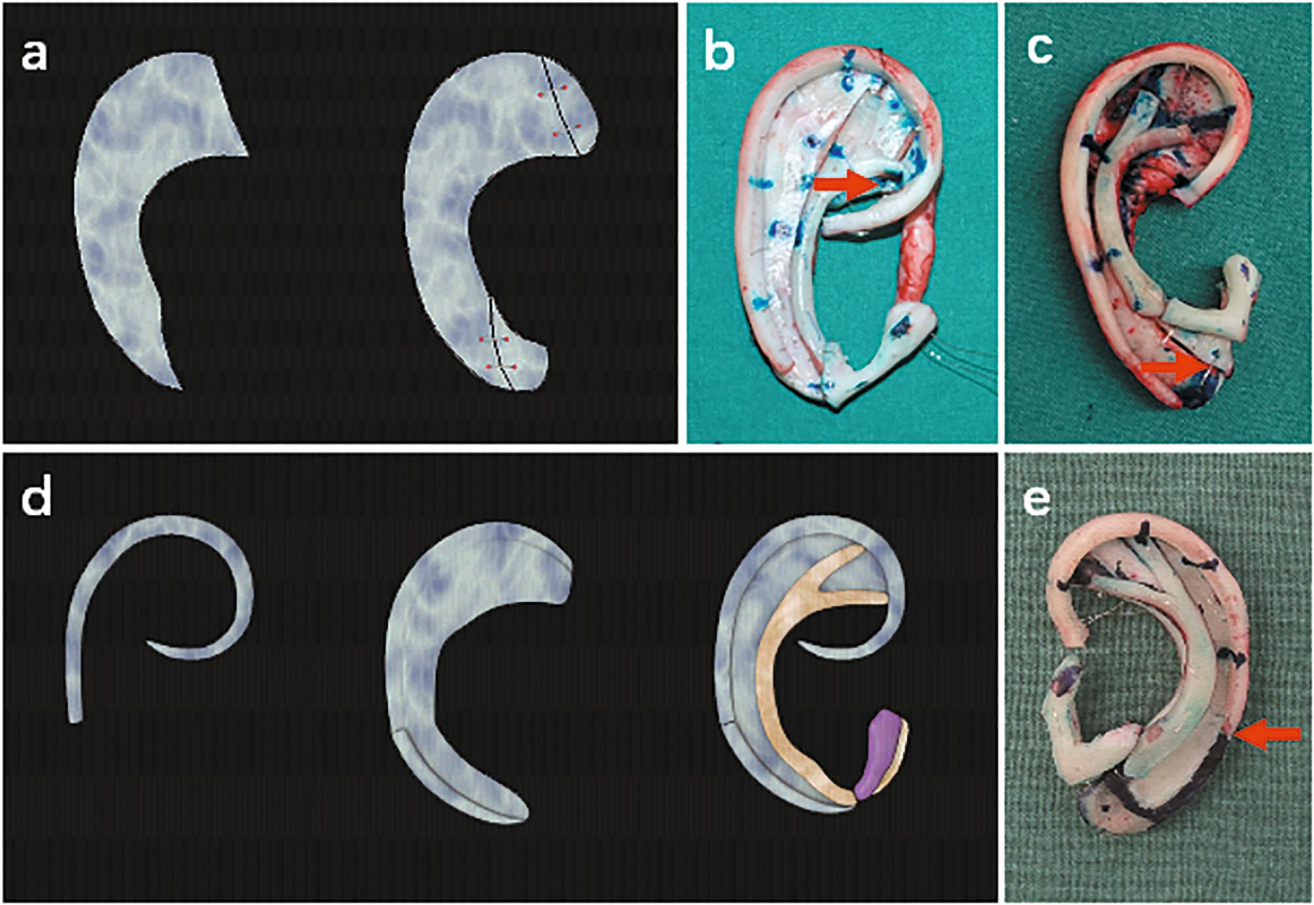
Schematic representation of the base frame fabrication with narrow cartilage (**a**–**c**) and a thick base frame fabrication from the relatively short eighth rib cartilage (**d**,**e**). (**a**) A piece of cartilage with the same thickness as the base frame is added laterally to increase the width of a narrow sixth or seventh cartilage. (**b**) The narrow sixth cartilage is widened. The red arrow shows the connection point. (**c**) The narrow lower part of the seventh cartilage is broadened. The red arrow shows the connection point. (**d**) The edge of the lower part of the base frame is carved as an extension of the helix body. (**e**) Completed three-dimensional framework. The red arrow shows the connection point.

In some cases, we found that the sixth rib cartilage was slightly narrow compared to the template from the contralateral side. Thus, to stably support the helix and maintain a proper width of the upper part of the auricle, laterally adding a piece of cartilage to broaden its width is recommended (Fig. 3a,b, yellow arrows in Fig. 5c,d, type C1).

In some adult and adolescent patients, for example, when the sixth and seventh rib cartilages are very thick, commonly > 5 mm, and the eighth rib cartilage is simultaneously relatively short, we can carve the edge of the lower part of the seventh rib cartilage to be an extension of the helix body (Fig. 3d,e, purple arrow in Fig. 5a, type C2). In this way, in helix fabrication, there is no need to extend the short eighth rib cartilage using additional residual cartilage.

**Figure 4:**
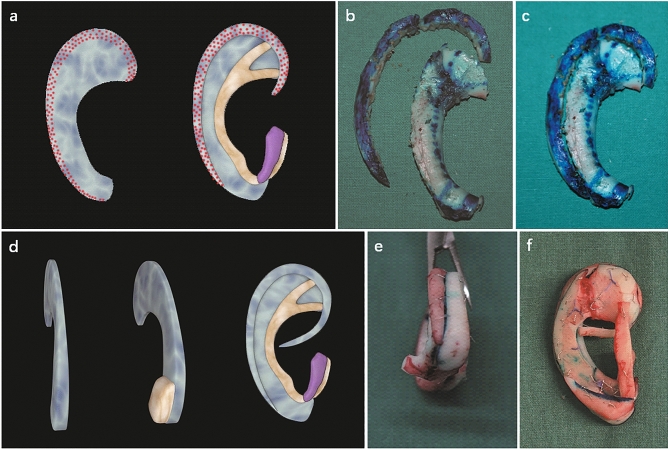
Helix fabrication with calcified or brittle cartilage (**a**–**c**) and schematic representation of the base frame fabrication in the anotia or some lobule-type patients whose residual tissue cannot be utilized as the lobule (**d**–**f**). (**a**) Schematic representation of the helix fabrication with calcified or brittle cartilage. (**b**) The outer edge of the base frame is cut off and used as the helix. (**c**) Completed framework ready for reconstruction. (**d**) Note that the lower part of the base frame is kept as thick as possible. If necessary, a block of cartilage is added at the bottom of the lower part of the base frame to protrude the contour of the ear lobule. (**e**) Lateral view of the completed three-dimensional framework. (**f**) A block of cartilage is added at the bottom of the lower part of the base frame to protrude the contour of the ear lobule.

Occasionally, we may encounter rib cartilage with special characteristics in some adult patients whose cartilage is calcified or too brittle. Such cartilage is very difficult to sculpt and is liable to fracture during fabrication or even after the operation. Therefore, in these cases, we cut off the outer edge of the base frame and used it as the helix (Fig. 4a–c, type C3). If the outer edge of the framework body is separated at the synchondrosis, then a connection procedure with stainless steel wire is necessary, as described above.

### Part D base frame and tragus–antitragus complex fabrication

**Figure 5:**
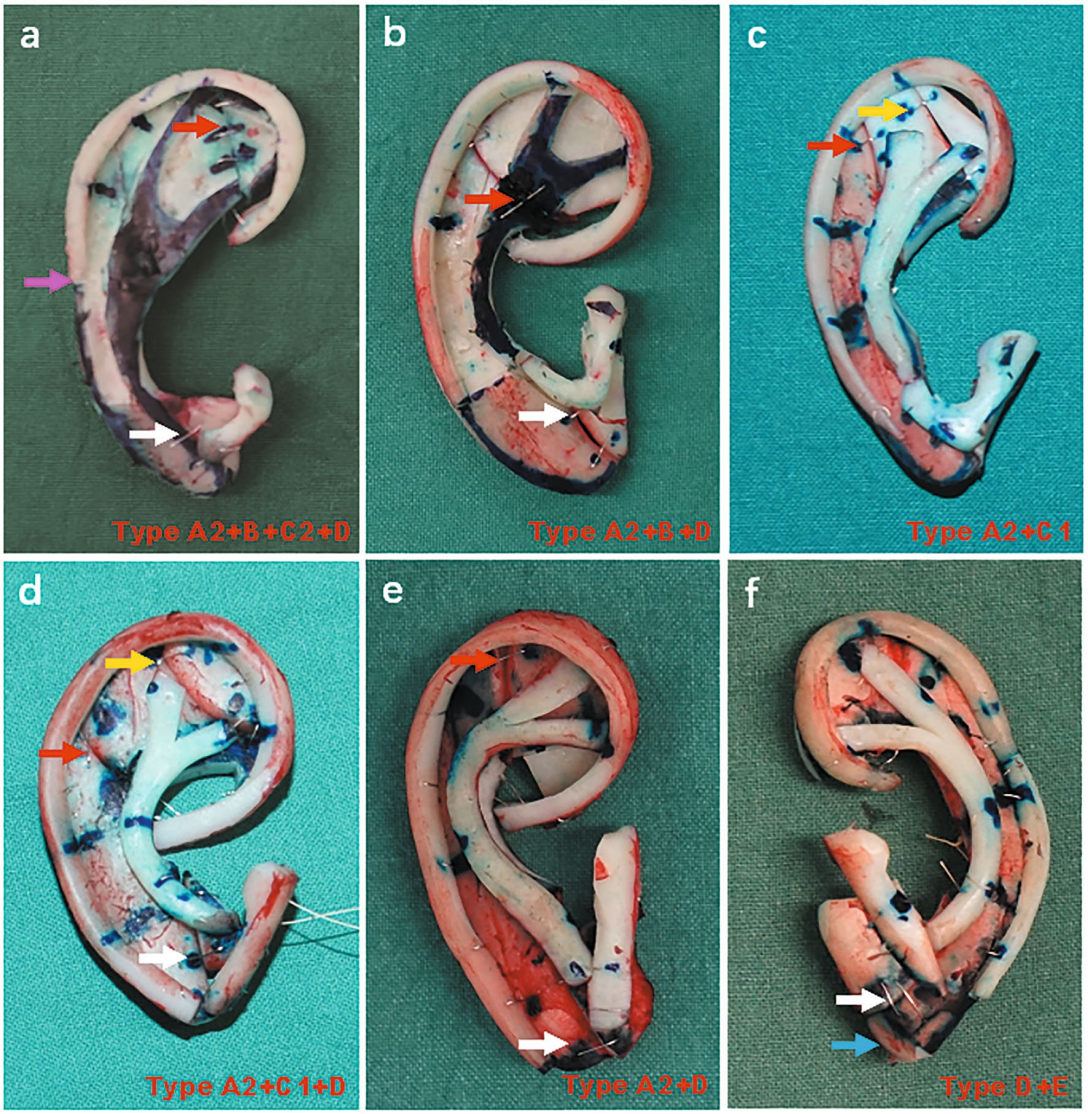
The base frame fabrication in different types. Red, yellow, purple and white arrows show the connection points in type A2, C1, C2 and D, respectively. The blue arrow shows the thickened lower part of the base frame. (**a**) Type A2 + B + C2 + D. The separated upper part is connected and the Y-shaped antihelical complex carved directly from the base frame. The edge of the lower part of the base frame is carved as an extension of the helix body and the narrow lower part of the seventh cartilage is broadened. (**b**) Type A2 + B + D. The separated synchondrosis is connected and the Y-shaped antihelical complex carved directly from the base frame. The narrow lower part of the seventh cartilage is broadened. (**c**) Type A2 + C1. The separated synchondrosis is connected, and the narrow sixth cartilage is widened. (**d**) Type A2 + C1 + D. The separated synchondrosis is connected, and the narrow sixth and seventh cartilages are widened. (**e**) Type A2 + D. The separated synchondrosis is connected, and the narrow lower part of the seventh cartilage is broadened. (**f**) Type D + E. The lower part of the base frame is widened and thickened.

The tragus–antitragus complex defines the width of the reconstructed auricle. However, if the seventh rib cartilage is comparatively narrow after reference to the template, we advocate adding a piece of cartilage laterally to widen it (Fig. 3a,c, white arrows in Fig. 5a,b,d–f, type D). By doing so, the tragus–antitragus complex could be placed in the proper position.

### Part E base frame and ear lobule fabrication

In anotia or some lobule-type patients, the residual tissue is limited in quantity or misplaced and cannot be utilized as the lobule. Since no remnant skin can be transposed to an appropriate position, the lower part of the base frame should be kept as thick as possible. If necessary, a cartilage cube can be fixed at the bottom of it to protrude and form the contour of the ear lobule (Figs. 4d–f, 5f, type E).

## Results

Follow-up ranged from 6 to 48 months, with a median of 10 months. All the patients were interviewed using a questionnaire during their follow-up session or over the telephone as described in our previous reports^11–13^. We found that 90% of the patients responded at follow-up, and 76% of them were satisfied with the cosmetically refined auricle with harmonious integrity (Table 3). Four patients categorized in Part A and one patient categorized in Part B complained about a heavy-looking framework after the second stage of the operation because of the thick base frame. Three patients categorized in Part C and two patients categorized in Part D complained about exposure of the steel wire at the junction. One adult patient categorized in Part E complained about the rigid appearance of the ear lobe. There were no pneumothoraxes or infections in this series. The rates of complications were steel wire extrusion (1.4%), flap venous congestion (2.3%) and skin necrosis/cartilage exposure (1.7%). With proper therapeutic treatment, the structure of the cartilage graft was preserved to a maximum extent without any obvious deformity.

**Table 3:** Patient characteristics of different types of base frame fabrication in microtia reconstruction.

Type	No. of patients (%^a^)	No. of response at follow-up (%^b^)	No. of satisfaction response at follow-up (%^c^)
A1	57 (16.19)	53 (92.98)	41 (77.36)
A2	158 (44.89)	143 (90.51)	109 (76.22)
B	3 (0.85)	3 (100.00)	3 (100.00)
B + A2	4 (1.14)	3 (75.00)	2 (66.67)
B + D	2 (0.57)	2 (100.00)	1 (50.00)
B + D + A2	2 (0.57)	2 (100.00)	1 (50.00)
B + C2 + D + A2	1 (0.28)	1 (100.00)	1 (100.00)
C1	14 (3.98)	13 (92.86)	10 (76.92)
C1 + A2	11 (3.13)	10 (90.91)	8 (80.00)
C2	12 (3.41)	11 (91.67)	9 (81.82)
C2 + A2	9 (2.56)	8 (88.89)	6 (75.00)
C3	7 (1.99)	6 (85.71)	5 (83.33)
C3 + A2	6 (1.70)	6 (100.00)	5 (83.33)
D	15 (4.26)	12 (80.00)	7 (58.33)
D + A2	14 (3.97)	11 (78.57)	7 (63.64)
D + C1 + A2	4 (1.14)	3 (75.00)	2 (66.67)
E	23 (6.53)	21 (91.30)	18 (85.71)
E + A2	5 (1.42)	4 (80.00)	4 (100.00)
E + D	3 (0.85)	3 (100.00)	2 (66.67)
E + D + A2	2 (0.57)	2 (100.00)	1 (50.00)
Total	352	317 (90.06)	242 (76.34)

^a^No. of patients/total patients × 100%; ^b^No. of response/no. of patients × 100%; ^c^No. of satisfaction response/no. of response × 100%.

## Case reports

### Case 1

**Figure 6:**
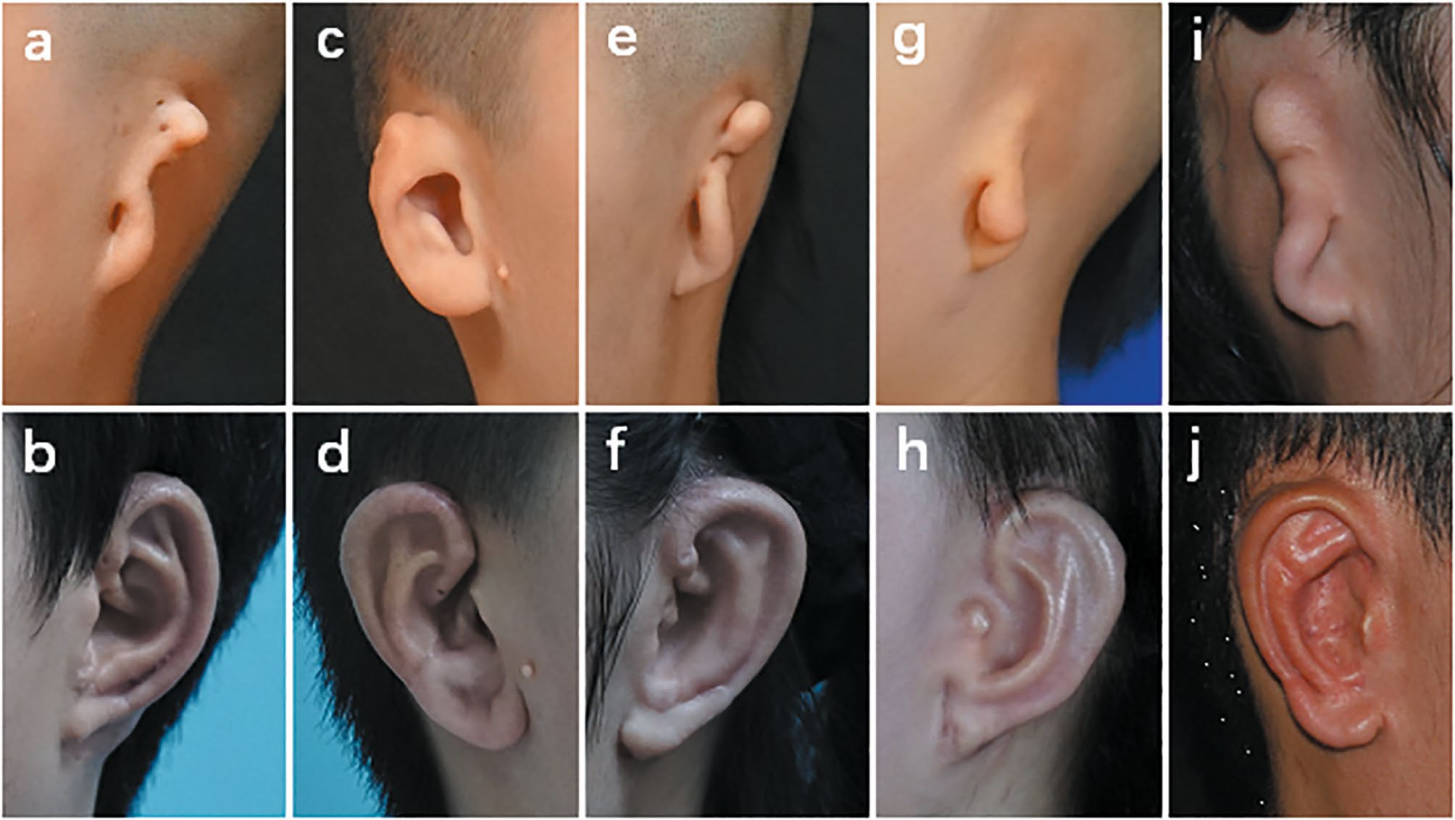
Case presentation. (**a**) Case 1. Preoperative oblique view of a 15-year-old boy who presented with lobule-type microtia. The base frame was constructed with completely integral synchondrosis and proper thick cartilage using our method of type A1. (**b**) Postoperative oblique view 1 year after ear elevation. (**c**) Case 2. Preoperative lateral view of an 11-year-old boy who presented with concha-type microtia. The narrow upper part of the base frame was broadened by adding a piece of cartilage with the same thickness as described in type C1. (**d**) Postoperative oblique view 8 months after ear elevation. (**e**) Case 3. Preoperative oblique view of a 14-year-old girl who presented with lobule-type microtia. The separate upper part of the base frame was fixed by stainless steel wire, and the narrow lower part was broadened by a piece of cartilage as described in types A2 and D. (**f**) Postoperative oblique view 8 months after ear elevation. (**g**) Case 4. Preoperative oblique view of a 10-year-old girl who presented with lobule-type microtia and whose residual tissue was limited in quantity and could not be utilized as the lobule. The earlobe was reconstructed together with the base frame as a whole as described in type E. (**h**) Postoperative oblique view 6 months after ear elevation. (**i**) Case 5. Preoperative oblique view of a 23-year-old man who presented with lobule-type microtia. The Y-shaped antihelical complex was carved directly from the base frame. The narrow lower part of the seventh cartilage was broadened, and the separated synchondrosis was connected, as illuminated in type A2 + B + D. (**j**) Postoperative oblique view 48 months after ear elevation.

The patient was a 15-year-old boy with lobule-type microtia on the left side (Fig. 6a,b). The base frame was fabricated as an integral whole using the technique described in type A1. The postoperative results 1 year after surgery were favourable. The auricle showed a natural contour, approximating the shape of the normal side.

### Case 2

Case 2 was an 11-year-old boy with concha-type microtia on his right side. The narrow upper part of the base frame was widened using the method described in type C1 (Fig. 6c,d). The framework was stable, and no breakage or incisure occurred when he was followed up at 8 months after the ear elevation. The reconstructed auricle appeared harmonious with a satisfactory shape.

### Case 3

The patient in this case was a 14-year-old girl with lobule-type microtia on the left side. The separate upper part of the base frame was fixed by a stainless steel wire, and the narrow lower part was widened by adding a piece of cartilage as described in types A2 and D (Fig. 6e,f). Over an 8-month follow-up period, the reconstructed auricle acquired a natural and clearly refined contour.

### Case 4

The patient was a 10-year-old girl with lobule-type microtia on her left side (Fig. 6g,h). The residual tissue was limited in quantity and could not be utilized as the lobule. Thus, the earlobe was reconstructed using the method demonstrated in type E. It appeared smooth and natural with clearly defined morphologic features 6 months postoperatively.

### Case 5

Case 5 was a 23-year-old man with lobule-type microtia on his right side (Fig. 6i,j). The Y-shaped antihelical complex was carved directly from the base frame. The narrow lower part of the seventh cartilage is broadened, and the separated synchondrosis is connected, as illuminated in type A2 + B + D. The auricle achieved satisfactory results over a 48-month follow-up period.

## Discussion

In auricular reconstruction, more attention has been given to the protruding subunits, such as the helix, antihelix, and tragus–antitragus complex. However, it is easy to overlook the significance of the base frame in framework fabrication. The base frame mostly supports the width and length of the auricle and needs to be a reliable support for the protrusive structures fixed on it and integrate into them as an indivisible entity. A framework with a solid foundation can effectively resist persistent contraction of the skin flap and maintain a harmonious appearance. Thus, the successful fabrication of the base frame is of great importance to the overall aesthetics of the auricle^14,15^. However, no report has systematically elaborated on the fabrication of base frames with various sizes and different characteristics.

We find that when the synchondrosis of the two ribs is firmly connected or even integral as a whole, it is more convenient to construct the base frame appropriately, as elaborated in type A. Concerning the thickness of the skin flap, the base frame needs to be reduced by 1–2 mm in diameter compared to the template from the normal side. Meanwhile, when the base frame is sculpted to an appropriate thickness, it is more likely to achieve the appearance of a delicate reconstructed auricle after the second stage of the operation. Otherwise, it would appear to be too cumbersome if the base frame is made too thick during the first stage.

Considering the integral harmony, we think it appropriate to keep the average thickness of the base frame between 4 and 5 mm. Moreover, the dorsal part of the base frame should be sculpted as smoothly as possible; otherwise, it would appear to be too sharpened at the edge and inevitably negatively impact the survival of the fasciae and skin during the second stage of the operation. Taking these steps will maintain the stability of the framework to the utmost degree and will present a delicate rather than a cumbersome contour at follow-up.

In many cases, we may encounter separate sixth and seventh costal cartilages, which will obviously influence the stability of the framework. To reinforce the framework and lessen the possibility of distortion, it is necessary to fix the divided cartilages together with nylon sutures or steel wires^16^. We prefer to use stainless steel wire because it is more reliable than nylon sutures for resisting the tensions in different directions. It is worth noting that the degree of wire tightness is relevant to the width of the framework. That is, we should screw the steel wires properly by referring to the template from the normal side. Otherwise, the base frame will be inevitably narrowed if the wires are screwed too tightly and if the size of the template is not considered. To lessen the exposure of the steel wires, we have been using wires that are 0.20 mm in diameter. It is convenient to cut and remove the comparatively thinner wires several months later at follow-up or during the second stage.

Several months after the first stage of the operation, proper occlusion occurs between the skin and framework. During the second stage, we may even find a complete membrane around the base framework. That is, the whole framework has obtained reliable nutrients from the blood supply and is quite stable several months later. Therefore, it is safe to remove the wires without experiencing a regression of the framework.

To conceal the notching of the synchondrosis and to prevent a staircase effect, the antihelix is often fixed on it. Meanwhile, we recommend carving a groove into the base frame to insert the Y-shaped antihelix complex and to add stability to the antihelix complex. Thus, this groove further enables a natural and smooth presentation of the antihelix, scapha and triangular fossa^17^. In some adults or adolescent patients with strong cartilage and no notch at the synchondrosis, we found that the Y-shaped antihelical complex could be carved directly from the base frame, as shown in type B. Nevertheless, we advocate not carving the subunit of the helix on the base frame simultaneously. The eighth rib is still the best selection for helix protrusion at the desired height^18^.

We think it important to maintain the proper width of the base frame, which is highly important for achieving an accurate position and integrity of the reconstructed auricle, especially the projected subunits such as the helix, tragus and antitragus. When the sixth rib is slightly narrow, as described in type C1, it will no doubt influence the stability of the front part of the helix, which is one of the most prominent parts of the ear and mainly supports the width of the auricle. On the other hand, if the seventh rib cartilage is not widened properly, the tragus would seem to be off-centre, as mentioned in type D. Moreover, the contour of the auricle would be top-heavy and inharmonious. Concerning this phenomenon, we advocate attaching a cartilage cube laterally to widen the base frame and then fix the relevant subunits in the proper position^19^.

Sometimes the base frame may assist with helix fabrication when it is thick enough and when the eighth costal cartilage is comparatively short, and thus the edge of the framework could be perfect for the extension of the short helix, as shown in type C2. Thus, neither additional residual cartilage nor the ninth rib is needed to connect and extend the helix. Occasionally, the base frame could be the source of the helix if the eighth rib is calcified or brittle. For this procedure, Brent sculpts the framework as one piece, not unlike a wood carving^2^. However, the cartilage is not as thick in Asian patients as in other populations. As suggested by Firmin^20^, great care must be taken to prevent fracturing when carving cartilage from the eighth rib in adult patients. It is necessary to pass the steel wire through the hole with the help of a burr to sculpt ossified or brittle cartilage. If it breaks, it should be replaced with a new rounded curve of the upper part of the helix formed by another piece of cartilage instead of fixing the broken curvature with steel wire. Otherwise, it may become deformed again at follow-up due to unstable fixation. In our experience, we obtain satisfactory results if we detach a stripe of cartilage from the outer edge of the base frame, normally from the sixth and seventh costal cartilages, and slide it up the base frame to augment the rim’s protrusion, similar to the helix. Steel wire fixation is necessary if the edge of the synchondrosis is separated.

As demonstrated in type E, when there is no utilizable remnant skin left to transpose to the appropriate position, we prefer to harvest cartilage from the ipsilateral side because the curve of the cartilage is helpful to protrude the lower part of the base frame, which has to be kept as thick as possible. If necessary, a block of residual cartilage could be added beneath the base frame to further protrude the subunit of the ear lobule, especially in those patients who also present with moderate-severe hemifacial macrosomia and an obvious depression around the auricular region. In addition, we recommend dissecting the skin flap from a larger region, keeping continuity of the subcutaneous vascular networks and maintaining the subcutaneous pedicle simultaneously, which achieves adequate looseness and facilitates blood supply to the skin flap^21,22^. In this way, the procedure ensures that the skin will accommodate the thickened base frame appropriately and reduce the risk of skin necrosis or cartilage exposure^23^.

In this series, the occurrence of flap venous congestion and skin necrosis/cartilage exposure may be related to the use of an inappropriate size for the subcutaneous pedicle and continuity destruction of the subcutaneous vascular networks due to uneven dissection of the flap. With proper therapeutic treatments, conventional measures such as flap punching and heparin gauze dressing^24^, hyperbaric oxygen therapy (HBOT)^25^ and salvage operations including a local random fascia flap or temporoparietal fascia flaps (TPFs) plus skin grafts^26^, the essential morphologic features of the reconstructed auricle can be maintained without obvious deformity.

During the process of base frame fabrication, several techniques may be applied simultaneously, as shown in Table 3. In addition to the general procedure, we find that the method of type A2 can be used alone or combined with the other techniques. Separated sixth and seventh costal cartilages are the most common cases we may encounter. Therefore, the primary task is to maintain the stability of the base frame. In addition, type D also occurs quite often. This reminds us that the narrow seventh rib cartilage is another important aspect we should consider. This part influences the entire symmetry of the framework. The contour of the auricle will be top-heavy and inconsistent if the seventh rib cartilage is not broadened properly. As shown in this table, we have found that two or three types of techniques need to be combined simultaneously in base frame fabrication. These combinations indicate that we may often encounter complicated conditions of the rib cartilage with different characteristics. To overcome such difficulties, the flexible application of the different types of techniques mentioned above in the proper combinations is necessary. We found that the response and satisfaction rate for Part D was slightly lower than that for the other Parts. To some extent, this could be informative for surgeons when making surgical decisions. In future work, we need to enhance patient satisfaction through better accentuation of the definition of the tragus–antitragus complex.

## Conclusions

The elaborate design and appropriate utilization of the costal cartilage for base frame sculpting is a crucial and fundamental procedure in microtia reconstruction, which is as important as the other projected structures. They contribute to achieving a natural appearance of the auricle with harmonious integrity at follow-up.
